# Genome-wide analysis reveals divergent patterns of gene expression during zygotic and somatic embryo maturation of *Theobroma cacao* L., the chocolate tree

**DOI:** 10.1186/1471-2229-14-185

**Published:** 2014-07-16

**Authors:** Siela N Maximova, Sergio Florez, Xiangling Shen, Nicolas Niemenak, Yufan Zhang, Wayne Curtis, Mark J Guiltinan

**Affiliations:** 1 Department of Plant Science and Huck Institute of Life Sciences, The Pennsylvania State University, University Park, PA 16802, USA; 2 Laboratory of Plant Physiology, Department of Biological Science, Higher Teachers’ Training College, University of Yaounde, Yaounde, Cameroon; 3 Department of Chemical Engineering, The Pennsylvania State University, University Park, Pennsylvania 16802, USA

**Keywords:** *Theobroma cacao*, Somatic embryogenesis, Zygotic embryogenesis, Embryogenesis, Microarray, Gene expression

## Abstract

**Background:**

*Theobroma cacao* L. is a tropical fruit tree, the seeds of which are used to create chocolate. *In vitro* somatic embryogenesis (SE) of cacao is a propagation system useful for rapid mass-multiplication to accelerate breeding programs and to provide plants directly to farmers. Two major limitations of cacao SE remain: the efficiency of embryo production is highly genotype dependent and the lack of full cotyledon development results in low embryo to plant conversion rates. With the goal to better understand SE development and to improve the efficiency of SE conversion we examined gene expression differences between zygotic and somatic embryos using a whole genome microarray.

**Results:**

The expression of 28,752 genes was determined at 4 developmental time points during zygotic embryogenesis (ZE) and 2 time points during cacao somatic embryogenesis (SE). Within the ZE time course, 10,288 differentially expressed genes were enriched for functions related to responses to abiotic and biotic stimulus, metabolic and cellular processes. A comparison ZE and SE expression profiles identified 10,175 differentially expressed genes. Many TF genes, putatively involved in ethylene metabolism and response, were more strongly expressed in SEs as compared to ZEs. Expression levels of genes involved in fatty acid metabolism, flavonoid biosynthesis and seed storage protein genes were also differentially expressed in the two types of embryos.

**Conclusions:**

Large numbers of genes were differentially regulated during various stages of both ZE and SE development in cacao. The relatively higher expression of ethylene and flavonoid related genes during SE suggests that the developing tissues may be experiencing high levels of stress during SE maturation caused by the *in vitro* environment. The expression of genes involved in the synthesis of auxin, polyunsaturated fatty acids and secondary metabolites was higher in SEs relative to ZEs despite lack of lipid and metabolite accumulation. These differences in gene transcript levels associated with critical processes during seed development are consistent with the fact that somatic embryos do not fully develop the large storage cotyledons found in zygotic embryos. These results provide insight towards design of improved protocols for cacao somatic embryogenesis.

## Background

*Theobroma cacao* L. (cacao) is a diploid tree grown in more than 50 tropical countries as a major cash crop that provides income to millions of small-holder farmers [1]. The fermented and dried seeds of cacao provide the basis for a multi-billion dollar cash crop of importance to the economic trade and social development of these regions. Cacao seeds consist primarily of embryonic cotyledons that form a highly invaginated tissue rich in oils, terpenes, proteins, starch and flavonoids.

Plant embryo development is initiated with the double fertilization of male and female gametes, followed by the process of zygotic embryogenesis (ZE), during which the formation of the embryo occurs by a complex program of lateral, radial and longitudinal growth [2,3]. Embryonic growth has been classified into globular, heart, torpedo, and mature stages of development [4] and proper progression through these developmental stages is controlled by a sophisticated regulatory network [2,3,5,6].

Despite its complexity, the embryogenic process can be artificially induced from somatic tissues in many plant species including cacao [5,7], in a process known as somatic embryogenesis (SE), first reported with carrot in 1958 [8]. During SE maturation, cells develop completely outside the maternal context into somatic embryos and by ‘conversion’, into whole morphologically intact plants [9]. In some plants, this complicated process can be initiated by simple manipulation of hormones, whereas in others, its efficiency is dependent on genotype and explant tissue type, and requires precise control of hormonal and environmental conditions. Extensive research has focused on the discovery of genes that control the highly complex regulatory mechanisms leading the initiation and regulation of embryogenesis [5,10-13]. Somatic and zygotic embryos follow the same overall pattern of development despite the fact that somatic embryo initiation is preceded by the dedifferentiation of somatic cells rather than formation of haploid gametes via meiosis as in sexual reproduction and occurs in the absence of endosperm and maternal ovary tissues [11,14]. In plant seeds, endosperm plays an important role in providing nutrients to the embryo during development and germination [3,15,16] and it has been shown to play a role in integrating the different components and genetic programs of seed development [17]. The main axis of cacao SEs morphologically resembles its zygotic counterpart with a bipolar structure and typical embryonic organs. However, during zygotic seed development, the embryonic cotyledons expand and accumulate large amounts of lipids, protein, carbohydrates and terpenes, while somatic embryos produce small underdeveloped cotyledons.

The SE protocol for cacao includes 4 major steps: primary somatic embryogenesis, secondary somatic embryogenesis, somatic embryo conversion to plantlets and plant acclimation [18,19]. SEs of cacao have been generated from many different genotypes and SE-plants have been grown under field conditions, and have demonstrated growth similar to plants propagated by seeds [18-23]. Although the system is sufficiently efficient to be utilized commercially, it includes several steps that are highly genotype dependent. One of the most inefficient steps of the procedure is somatic embryo conversion, which involves transition of mature somatic embryos into whole plants [24]. The conversion rates depend on many factors including genotype and success rates vary from 7 to 75% (author’s unpublished data).

In order to investigate the regulatory and gene expression networks involved during ZE and SE maturation in cacao, we characterized gene expression profiles using whole genome microarrays. Expression profiles from 4 zygotic and 2 somatic embryo developmental stages demonstrated major differences in expression of transcription factors, flavonoid biosynthesis genes and lipid biosynthesis genes during zygotic and somatic embryo maturation suggesting a molecular basis for the lack of full development of somatic embryo cotyledons. Understanding the genetic components regulating the SE developmental cascade could provide guidance towards controlling the somatic embryo maturation process to improve the efficiency and quality of plantlets produced through manipulation of the chemical and physical culture environment. Further, our results provide new knowledge of gene expression programs during zygotic embryo development in cacao, which provides insight into the basic biology of seed development of an important tropical tree crop.

## Results

### Overview and microarray analysis

**Figure 1:**
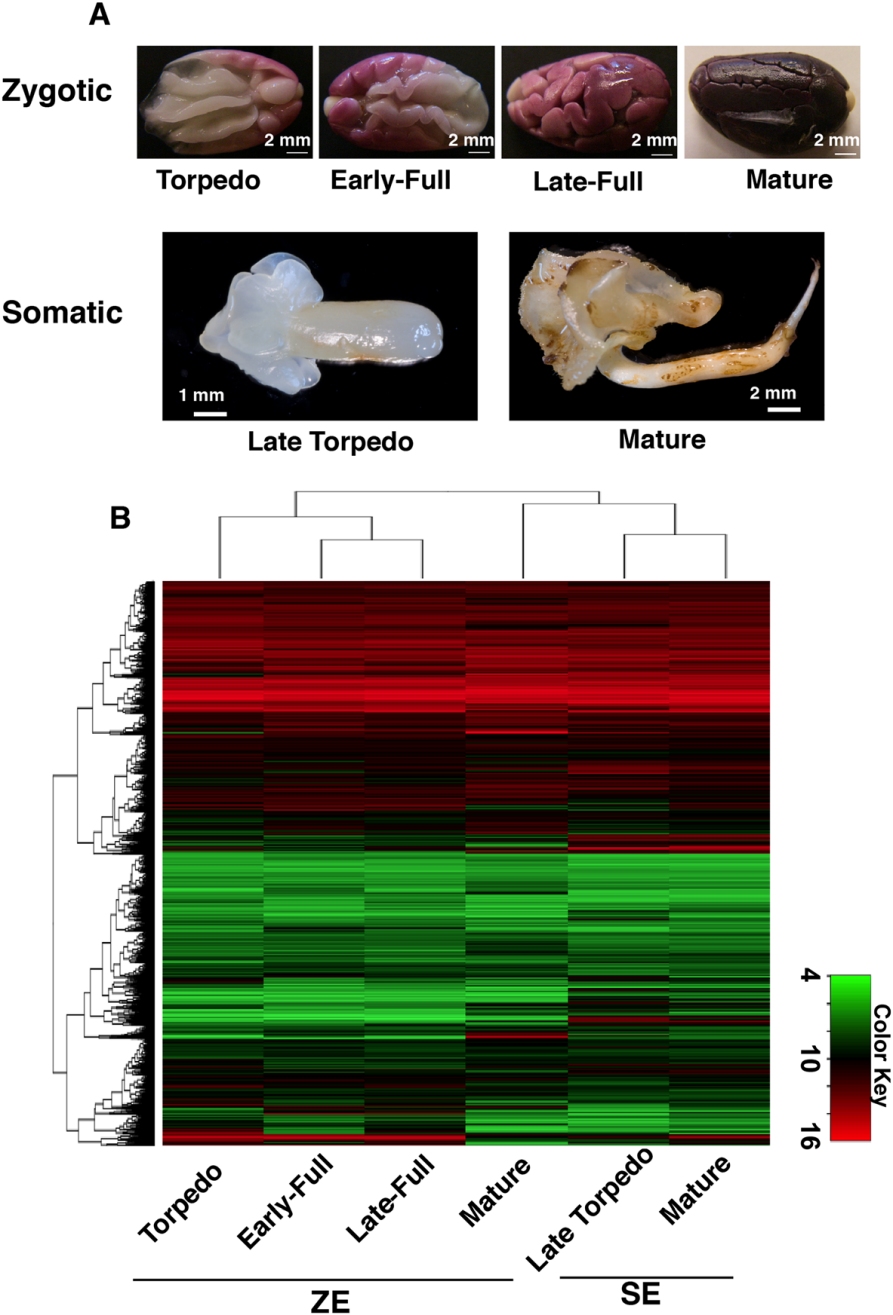
**Global gene expression analysis of cacao embryogenesis. (A)** Representative images of zygotic and somatic embryo stages used for RNA extraction. Zygotic Embryos at stages: Torpedo, Early-Full, Late-Full, Mature. Somatic Embryos at stages Late Torpedo and Mature. **(B)** Global gene expression Heatmap and Cluster analysis of cacao zygotic and somatic embryo samples. Cluster analysis on Y axis represents similarities in overall gene expression profiles while cluster analysis on the X axis (above) indicates the relatedness of gene expression profiles between the different tissue samples. The log_2_ of relative gene expression levels are depicted in color values indicated by the color key at bottom right.

Using the published cacao Criollo genome assembly V1.0 [1], a whole genome 12-plex Nimblegen expression microarray representing 28,752 genes was manufactured on which only 46 of the predicted cacao genes were not represented by at least one probe. To study cacao embryo development, total RNA was isolated from four biological replicates of six different tissue types and stages of embryo development: zygotic embryo tissues - torpedo (T-ZE), early-full (EF-ZE), late-full (LF-ZE) and mature (M-ZE) embryos; and somatic embryos - late torpedo (LT-SE) and mature (M-SE) stages. Representative images of embryos at these stages are depicted in Figure 1A. Twenty-four RNA samples were hybridized to a single chip simultaneously using two-color fluorescent labeling. After background subtraction and normalization, a heatmap was generated to represent the global dynamics of gene expression during zygotic and somatic embryo maturation (Figure 1B). Clustering analysis of the datasets indicated that the two SE developmental stages clustered closest to the profile of the mature developmental stage of ZE as might be expected.

### Dynamics of gene expression during zygotic embryo development

**Figure 2:**
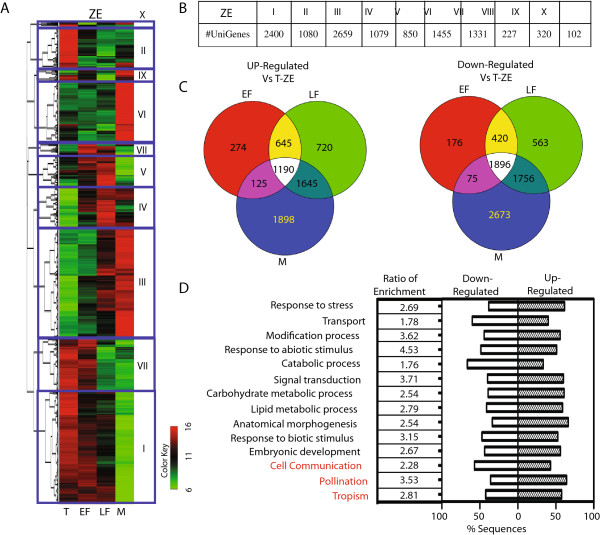
**Differential gene expression during zygotic embryo development (ZE). (A)** Heatmap of all differentially expressed genes during zygotic embryo maturation: Torpedo (T), Early-Full (EF), Late-Full (LF), Mature (M). All differentially expressed genes were grouped into 10 clusters using the K-means algorithm. **(B)** Number of differentially expressed genes in each ZE cluster. **(C)** Venn-diagram of differentially expressed genes in ZE. **(D)** GO enrichment analysis of all differentially expressed genes in ZE. Go categories in red indicate the genes enriched only in ZE group.

To identify differentially expressed genes during ZE maturation, a paired t-test analysis (*p < 0.01, adjusted Bonferroni correction*) was performed with cut-off of p < 0.01 and ≥ 2-fold change. 10,288 genes were identified to be either up or down regulated in this dataset (Additional file 1). The list includes all significant genes that were differentially expressed in at least one of the stages when compared to the T-ZE expression value. K-means clustering established 10 clusters representing different expression patterns containing between 102 and 2659 significantly regulated genes (Figure 2A and B). Relative to the T-ZE, large sets of genes were regulated uniquely in each stage and sets of genes overlapping between all of the stages were also observed. The total number of differentially expressed genes (relative to T-ZE) increased sequentially through the progression of embryogenesis (Figure 2C).

To further explore the biological pathways involved in ZE maturation, Gene Ontology (GO) terms were assigned to the significantly regulated genes, revealing that 5700 genes had annotations associated with various biological processes (Figure 2D). We explored the enrichment of GO categories in the significantly regulated gene sets as compared to their proportions found in the entire genome. Fourteen different GO categories were significantly enriched, with responses to abiotic and biotic stimulus and response to stress being among the most highly enriched (Figure 2D, Additional file 2).

### Dynamics of gene expression during somatic embryo maturation

We compared the gene expression profiles of 2 advanced somatic embryo developmental stages (LT-SE and M-SE) using *p* < 0.01 and ≥ 2-fold change filters and detected significant differences in gene expression levels. The significant genes (4,420) distributed into 4 major K-means clusters (Additional files 3 and 4). GO enrichment analysis indicated that genes associated with “Transport”, “Catabolic process”, “Signal transduction” and “Response to stress” where enriched during transition from LT-SE stage to M-SE stage (Additional file 4B). All but three of these GO categories were also enriched in ZE development (Figure 2D). Taken together, our results indicated that zygotic and somatic embryo maturation in cacao largely share similar transcriptome profiles, with some unique differences including genes involved in cell communication, pollination, tropism in ZE (Figure 2D), and in cell cycle, secondary metabolism, cellular homeostasis in SE (Additional file 4).

### Comparison of zygotic and somatic embryo maturation

**Figure 3:**
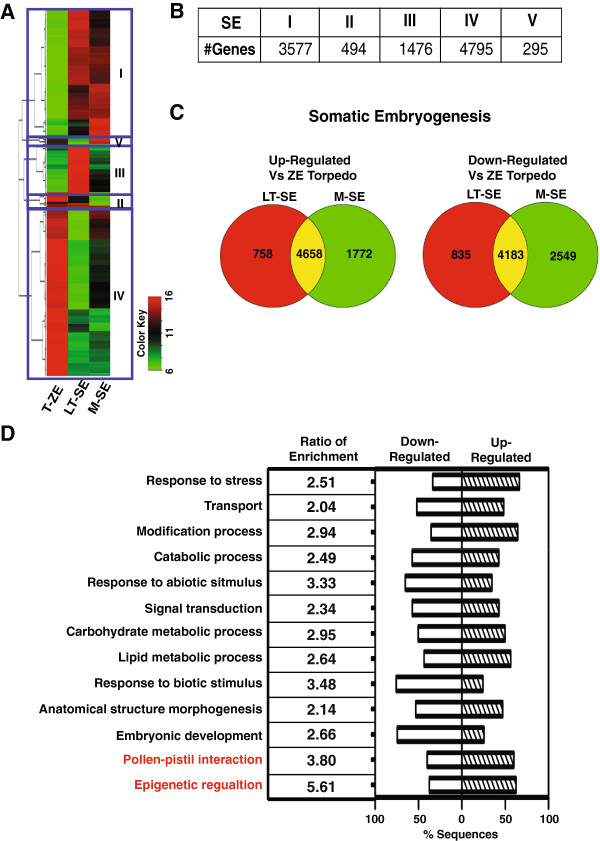
**Cluster analysis of differentially expressed genes during somatic embryo (SE) maturation using torpedo zygotic embryo developmental stage as a reference. (A)** Heatmap of all SE differentially expressed genes, stages Late Torpedo (LT-SE) and Mature (M-SE) compared to Torpedo Zygotic Embryo stage (T-ZE). All differentially expressed genes were grouped into 5 clusters using K-means algorithm. **(B)** Number of differentially expressed genes in each SE cluster. **(C)** Venn-diagram of differentially expressed genes in SE. GO categories labeled in red indicate the genes enriched only in SE group. **(D)** GO enrichment analysis of all differentially expressed genes in SE. GO categories labeled in red indicate the genes enriched only in SE group.

**Figure 4:**
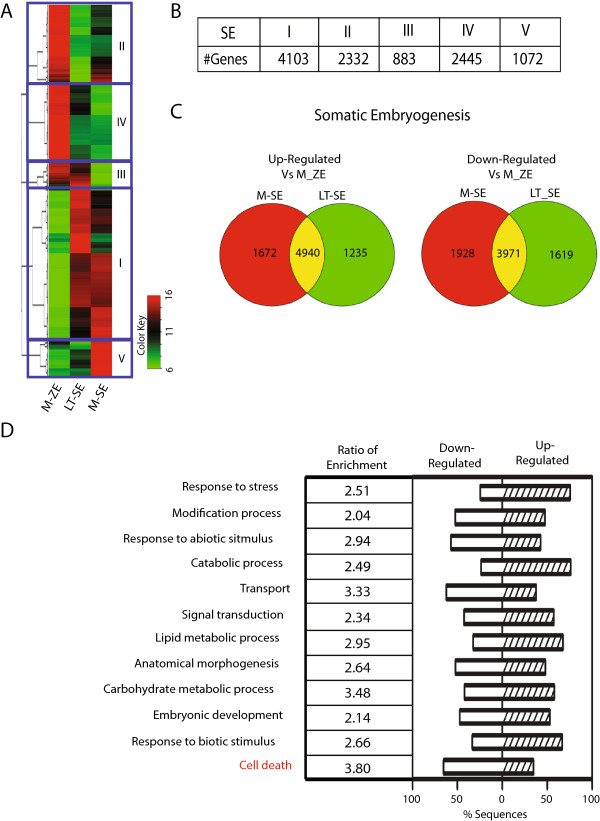
**Cluster analysis of differentially expressed genes during somatic embryo (SE) maturation using mature zygotic embryo developmental stage as a reference. (A)** Heatmap of all SE differentially expressed genes, stages Late Torpedo (LT-SE) and Mature (M-SE) compared to Mature Zygotic Embryo stage (M-ZE). All differentially expressed genes were grouped into 5 clusters using K-means algorithm. **(B)** Number of differentially expressed genes in each MSE cluster. **(C)** Venn-diagram of differentially expressed genes in MSE. **(D)** GO enrichment analysis of all differentially expressed genes in SE. GO categories labeled in red indicate the genes enriched only in SE group.

To perform a comparative analysis, we conducted two different cluster analyses contrasting the two somatic embryo stages with either T-ZE or M-ZE zygotic stages (Figures 3 and 4). A large number of differentially regulated genes were detected in the SE/T-ZE comparison that grouped into five clusters (Figure 3B and Additional file 5). The number of differentially expressed genes between T-SE and T-ZE (10,434) is comparable to the number of differentially expressed genes identified in the developmental transition from T-ZE to M-ZE (11,258 genes, Figure 2C), which illustrates the dramatic differences between torpedo stage somatic and zygotic embryos.

Fewer differences in gene expression were detected in the comparison of SE/M-ZE (4,708 up-regulated and 4506 down-regulated, Figure 4 and Additional file 6). Of these, 1949 genes were assigned to both the up and down regulated groups since their expression was either higher or lower depending on the SE stage. Consistent with the initial clustering of M-ZE with the two SE stages, the number of differentially expressed genes was 30% lower than when compared to T-ZE.GO annotation enrichment analysis was performed to explore the differences between zygotic and somatic embryo gene expression profiles (Figures 3D and 4D). For both comparisons, the most widely represented enriched GO terms were associated with response to abiotic and biotic stimulus, metabolic and cellular processes. We also detected enrichment of “Pollen-pistil interaction” and “Epigenetic regulation” genes only in the T-ZE specific comparison (Figure 3D). In the mature zygotic embryo comparison, “Cell death” was uniquely enriched, suggesting a difference in the regulation of apoptosis during the different modes of embryo maturation (Figure 4D).

### Functional classification of differentially expressed genes in zygotic and somatic embryo maturation

In contrast to the 10 clusters of genes with similar gene expression profiles identified by K-means clustering methods (Figure 2A and Additional file 7) when comparing ZE stages, five expression clusters of genes were identified when comparing the two SE developmental stages to T-ZE (Figures 3 and 4 and Additional file 8). To obtain a quantitative assessment of the biological processes involved in the different clusters, GO enrichment analysis was performed (Additional files 7 and 8). The dominant functional GO term annotations in each cluster were associated with “Metabolic and Cellular processes”. The down-regulated cluster I of the ZE group contained a proportionately greater number of genes in signaling and biological regulation (5%). In SE, the down-regulated cluster IV contained more “Cellular process” genes, whereas more “Metabolic process” genes were obtained in up-regulated clusters (Additional file 8). These results suggest that up-regulation of metabolic processes likely play an important role in somatic embryo maturation. This led us to further examine the specific classes of genes associated with seed development, namely, transcription factors and major enzymes of the major seed metabolic pathways.

### Expression profiles of transcription factor genes and genes associated with major seed-related metabolic pathways

**Figure 5:**
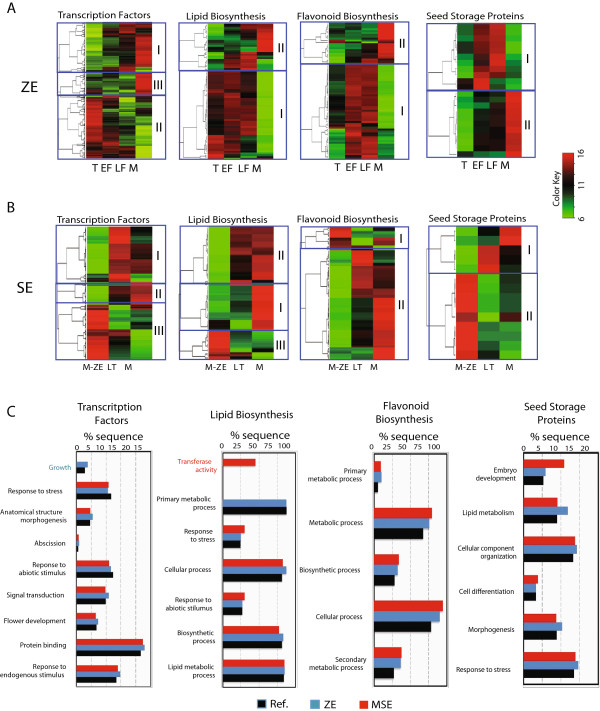
**Expression profiles of transcription factor genes and genes associated with major seed-related metabolic pathways during zygotic and somatic embryo maturation. (A)** Heatmap of all differentially expressed transcription factors, lipid biosynthesis, flavonoid biosynthesis and seed storage proteins genes during zygotic embryo maturation: Torpedo (T), Early-Full (EF), Late-Full (LF), Mature (M). The differentially expressed genes were grouped into different clusters using the K-means algorithm; **(B)** Heatmap of all differentially expressed transcription factors, lipid biosynthesis, flavonoid biosynthesis and seed storage proteins genes during somatic embryo maturation: Late Torpedo (LT-SE) and Mature (M-SE) compared to Mature Zygotic Embryo stage (M-ZE). The differentially expressed genes were grouped into different clusters using the K-means algorithm. **(C)** Results from GO enrichment analyses of all differentially expressed transcription factors, lipid biosynthesis, flavonoid biosynthesis and seed storage proteins genes in ZE and SE. Comparison was done using blast2go software. GO category in blue represents only genes enriched in the ZE group while red presents enrichment only in SE.

Based on the accumulated knowledge gained with model plant systems, four functional groups of genes were selected for analysis: transcription factors, lipid biosynthesis, flavonoid biosynthesis and seed storage proteins (Figure 5A and B). We investigated all genes in these classes that were differentially expressed during zygotic embryo maturation and based on the observation that the two SE developmental stages clustered closest to the M-ZE, we selected to compare the somatic embryo mature stage relative to mature zygotic embryos.

### Transcription factor genes

Based on a publication by Mitsuda and Ohme-Takagu [25] a list of plant transcription factors was generated as queries to perform tBlastX homology searches within the cacao genome. The homology analysis identified 736 cacao genes potentially coding for transcription factors (Tc-TF), of which 463 and 417 were differentially expressed during ZE and SE maturation respectively (Additional files 9 and 10). 54% (303) of these were differentially expressed during both, ZE and SE maturation. K-means clustering of the differentially expressed, in at least one stage, Tc-TF genes resulted in 3 subclusters in both SE and ZE development (up-regulated clusters I and III and down-regulated clusters II, Figure 5A and B).

GO enrichment analysis indicated only minor differences in representation of the major functional classes of transcription factors (Figure 5C). Functional categories were also assigned to significant Tc-TF genes using MapMan software [4] (Additional file 11). The MapMan analysis revealed 26 transcription factors in the “Ethylene metabolism and Ethylene response” categories, of which 19 showed significantly higher expression levels in SE compared to ZE. The majority of these cacao genes are related to Arabidopsis genes that are rapidly induced by ethylene, belonging to the ERF/AP2 family, which control the synthesis of proteins that mediate physiological and developmental responses to ethylene [26]. This could indicate that ethylene, a known stress response hormone, is accumulating in our culture system and this could be triggering downstream ethylene mediated stress response unfavorable to embryo development.

**Figure 6:**
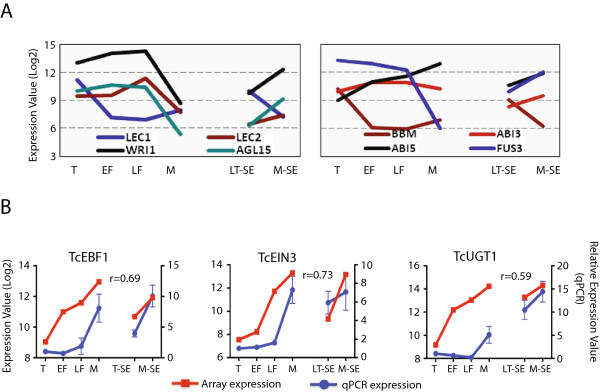
**Expression patterns of *T. cacao *selected embryogenesis and stress related genes regulated during cacao zygotic (ZE) and somatic (SE) embryo maturation. (A)** Expression patterns of 8 selected transcription factor genes described to be involved in embryogenesis in other plant species. Data represents normalized expression values from microarray analysis in all developmental stages of ZE (T, EF, LF and M) and SE (LT-SE and M-SE). **(B)** Three ethylene-signaling genes were randomly chosen for qRT-PCR analysis and patterns were compared to microarray results. Correlation coefficients (r) between microarray and qRT-PCR are indicated.

A targeted subset of cacao TF genes similar to genes in other species previously implicated to play role in embryogenesis [5,13,27-30] were examined including: *TcLec1* (*Tc07g001180*), *TcWRI1* (*Tc10g012790*), *TcLec2* (*Tc06g015590*), *TcAGL15* (*Tc01g040120*), *TcBBM* (*Tc05g019690*), *TcABI5* (*Tc05g008870*), *TcABI3* (*Tc01g024700*) and *TcFUS3* (*Tc04g004970*) (Figure 6A). Genes that had dramatically reduced expression values in M-ZE compared to the other ZE stages included *TcLec1*, *TcLec2*, *TcWRI1*, *TcAGL15*, and *TcFUS3*. Surprisingly, except for *TcLec1*, the expression of these genes is higher in M-SE samples compared to the less mature LT-SE. *TcBBM* and *TcABI5*, showed a slight decrease in expression from LF-ZE to M-ZE. *TcBBM* however, exhibited the opposite behavior in the SE stages (Figure 6A, right panel). These results reveal a large variation in the expression of transcription factors associated with embryogenesis between zygotic and somatic embryos and this likely contributes to the dramatic differences in morphological appearance of these structures (Figure 1A).

### Differential regulation of fatty acid biosynthesis pathway during maturation of zygotic and somatic cacao embryos

Cacao seeds contain about 50% cocoa butter, which is the one of the most valuable fats for industrial use, and the accumulation of fats in the cotyledon is closely related to seed development and maturation [31]. We investigated the expression changes of 84 cacao genes (Tc-FA) that were previously identified and annotated to be potentially involved in fatty acid biosynthesis [1]. In the comparison of M-SE to M-ZE, 32 out of 40 differentially expressed genes were up-regulated in M-SE (Additional file 12). Functional characterization of the significant Tc-FA genes indicated that a large set of plastidial fatty acid biosynthesis genes were up-regulated in M-SE compared to M-ZE, suggesting up-regulation of the fatty acid metabolic pathway in mature SE tissues (Additional file 12). For example, genes involved in fatty acid biosynthesis initiation steps, such as *acetyl-CoA carboxylase* (*Tc08g009450; EC 6.4.1.2,* 3.6; fold up-regulated*), biotin carboxyl carrier protein of acetyl-CoA carboxylase 2* (*Tc04g010240; BCCP; EC 6.4.1.2*; 6.2 fold up-regulated), *biotin carboxylase* (*Tc00g000210; CAC2, EC 6.3.4.14*; 10.3 fold up-regulated) and several isoforms of *3-ketoacyl-CoA synthase* (*Tc00g015810* and *Tc04g024470*; *KCS*; 47 fold up-regulation) were up-regulated in M-SE compared to M-ZE, all of which control the carbon flux leading to *de novo* fatty acid biosynthesis. In addition, genes involved in determination of the fatty acid profile, were also up-regulated in the M-SE compared to M-ZE, including *stearoyl-ACP desaturase* (*Tc08g012550*; *FAB2*, *EC 1.14.19.1*), *oleoyl-ACP thioesterase* (*Tc01g022130*; *FatA*; *EC 3.1.2.14*), *plastidial β-ketoacyl-ACP synthase II* (*Tc09g006480*; *KASII*; *EC 2.3.1.179*) and *plastidial omega 3 desaturase* (*Tc05g002310*; *FAD7/8*; *EC 1.14.19*). Differences in the expression levels of these key enzymes could contribute to the higher degree of fatty acid unsaturation observed for mature somatic embryos as compared to zygotic seed tissue (Y. Zhang, unpublished data). It is worth noting that the dynamics of lipid accumulation could be contributing to the observed FA transcript dynamics: considerable accumulation of lipids has occurred prior to the selected mature zygotic embryo stage, but not the ‘mature’ somatic embryo stage.

### Differential regulation of flavonoid metabolism genes during maturation of zygotic and somatic cacao embryos

Flavonoids are ubiquitous in the plant kingdom and have many diverse functions including defense, UV protection and auxin transport inhibition [11,32-34]. The expression of 94 cacao genes (Tc-FB, [1]) homologous to known flavonoid biosynthesis genes were examined and of these, 49 and 52 were differentially expressed during ZE maturation and SE maturation respectively (Additional files 13 and 14). K-mean analysis was used to generate up and down-regulated gene clusters for both groups (Figure 5A and B). Thirty-nine of the 52 regulated genes in SE were also regulated in ZE.

The analysis also demonstrated that from the 52 differentially expressed genes in the SE/M-ZE comparison, 45 genes were expressed significantly higher in M-SE as compared to M-ZE, indicating more active secondary metabolism in M-SE. The functional categories assigned by MapMan supported the prediction that Tc-FB genes are indeed involved in the synthesis of secondary metabolites including flavonoids, phenylpropanoids and lignin, but additionally MapMan assigned 6 of the genes to the “Ethylene synthesis and degradation” category (Additional file 15), indicating possible co-regulation of the flavonoid and ethylene biosynthesis pathways. These included genes: *Naringenin, 2-oxoglutarate 3-dioxygenases* (*Tc01g033570, Tc03g022510, Tc01g001700*), *Leucoanthocyanidin dioxygenase* (*Tc03g026420*), a gene for Flavonol synthase (Tc08g010270), and Flavonol synthase/flavanone 3-hydroxylase (*Tc05g019560*). Five of these genes (excluding *Tc01g033570*) had higher expression in M-SE compared to M-ZE.

### Differential regulation of seed storage protein genes during maturation of zygotic and somatic cacao embryos

Embryo maturation in the majority of higher plants is associated with accumulation of large amounts of storage proteins, which is regulated by a combination of hormonal, genetic and metabolic controls [35,36]. Based on a publication by Higashi [37] a list of seed storage proteins in *Arabidopsis* was generated as queries to perform tBlastX homology searches within the cacao genome. In our analysis, we selected 37 cacao genes coding for potential seed storage proteins (Tc-SSP). The LIMMA test identified 13 and 9 of these genes differentially expressed during ZE and SE maturation respectively (Figure 5A and B, Additional file 16). The K-mean analysis generated up-regulated (cluster II) and down-regulated cluster I for the ZE group, with an additional distinction of ‘Low-’ and ‘High-’ expressed gene clusters for the SE group (clusters I and II respectively). The 13 differentially expressed genes during ZE included a vicilin gene, a putative gene for the sweet protein mabinlin, 3 genes coding for 21-kDa trypsin inhibitor proteins, and 7 genes encoding for *Late Embryogenesis Abundant Proteins* (*LEA*) (Additional file 16). Significant genes identified during SE maturation compared to M-ZE included 2 of the same 21-kDa Trypsin inhibitor proteins genes, the expression of which was higher in M-SE compared to M-ZE (11.77 and 2.08 folds). Six of the 7 ZE significant genes encoding for LEA proteins were also differentially expressed during SE. During ZE maturation the expression of 6 of the 9 differentially expressed *LEA* genes increased with development reaching their highest expression values at the final M-ZE stage; the remaining 3 *LEA* genes demonstrated the opposite pattern with reduced M-ZE expression. By comparison, the expression values of the 6 differentially regulated LEA proteins were significantly lower during SE compared to M-ZE (Additional file 16). Although the expression of all 6 LEA genes increased from LT-SE to M-SE stages, these results indicated that the two SE stages are perhaps earlier in development compared to M-ZE.

An interesting observation was that the expression of the gene *Tc01g005060* was significantly lower in M-SE compared to M-ZE. This gene is nearly identical to the Arabidopsis *ABA1* gene, which encodes a zeaxanthin epoxidase critical for the first step in the synthesis abscisic acid (ABA). Since ABA is known to be important for the induction of genes involved in the synthesis of storage compounds and the maturation of embryos, this also suggests that the SE samples were at an earlier stage of development compared to the ZE samples.

### Validation of microarray data by RT-qPCR

To validate the microarray results, RT-qPCR analysis was performed using 10 randomly selected significantly regulated genes. Gene expression of samples from M-ZE and LT-SE were compared using the same RNA extractions used for the microarray hybridizations. The 10 genes demonstrated similar expression patterns in the microarray and the RT-qPCR analysis (Additional file 17). Although the r^2^ value of 0.54 suggests only a modest correlation in quantitative absolute values, in all but 1 of the ten genes tested, the relative differences in expression levels between ZE and SE samples were the same for the microarray and RT-qPCR data. Thus we consider the microarray data to adequately measure the relative gene expression changes, and the RT-qPCR data to more accurately quantify the precise quantitative differences.

Considering the observation that a large number of TF genes involved in ethylene signaling appear to be regulated during both ZE and SE development, we also additionally verified the expression patterns of three selected genes involved the ethylene signaling pathway: *TcEIN3-binding F-box protein 1*, (*TcEBF1, Tc09g011440*), *TcETHYLENE INSENSITIVE 3* (*TcEIN3, Tc09g033150*) and *TcIndole-3-acetate beta-glucosyltransferase* 2 (*TcUGT1, Tc02g020270*). Average expression values from microarray and RT-qPCR data were plotted for each developmental stage. A clear correlation was observed between the expression values of both analyses for all three genes (Figure 6B).

## Discussion

Development programs in plants are highly complex and require the interaction of many inter-related molecular processes, the activities of which need to be finely controlled and coordinated. Our whole genome expression analysis reveals this complexity as it relates to the mechanisms of embryo maturation in cacao. Roughly 36% of the nearly 29,000 genes are differentially expressed during zygotic embryo maturation with approximately equal numbers of up- and down-regulated genes. The number of significant genes increased with maturation, reaching 11,258 genes in mature zygotic embryos. These results indicate that cacao zygotic development is characterized by a significant shift in gene expression. This is consistent with studies of Arabidopsis where 54.9% (~14,000 unique mRNAs of 25,498 total) were differentially expressed during the various embryo developmental stages [1,3]. In the current study, in both zygotic and somatic embryogenesis, we observed a large number of differentially regulated genes that encode for transcription factors (TFs). This is consistent with the findings of Gliwicka et al., 2013, whom explored the changes of gene expression during somatic embryogenesis of Arabidopsis [38]. They observed 729 TFs whose expression changed during SE. Consistent with our results, they reported that many of the TF genes were down-regulated later in development of zygotic embryos, but continued high expression in later somatic embryo development. As in cacao, many of the TFs were annotated with functions related to plant development, phytohormones and stress responses.

A major difference between SEs and ZEs is the maturation of cotyledons, which in ZE results in large folded tissues containing flavonoids, lipids and seed storage proteins where in SE, results in only small, under-developed tissue. While gene expression profiles of SEs clustered most strongly with the mature ZE expression profile, a set of genes was also identified which is expressed strongly in SEs, suggesting that specific metabolic pathways might be associated specifically with SE and could help explain the difference in cotyledon development.

Further analysis of specific genes and pathways leading to seed development revealed that while many of the seed development specific genes are expressed in both types of embryos, many genes related to auxin and ethylene responses and to stress response were up-regulated in SEs. Many of these genes were expressed at higher levels in earlier stages of development and were not down-regulated in SEs as they are in normal seed development. Based on these results, we hypothesize that the lack of full cotyledon development in somatic embryos is a result of alterations in gene expression patterns due to accumulation of stress response signaling during the culture process. This could be triggered by stress resulting from sub-optimal media composition or environmental conditions.

### Regulation of hormone and flavonoid biosynthesis genes

Over 150 genes involved in auxin or ethylene metabolism and 352 genes related to biotic and abiotic stress are differentially expressed in SEs. This suggested that under *in vitro* conditions*,* ethylene signaling is up-regulated in cacao SEs, resulting in increased expression of ethylene-mediated stress response genes. A similar observation was reported in white and black spruce somatic embryogenesis where increases in ethylene signaling were implicated in abnormal embryo development [39,40]. It is plausible that increased stress response gene expression restricts cotyledon development in SEs as a result of reallocation of resources away from cotyledonary reserves.

Ethylene is also involved in the up-regulation of flavonol biosynthesis in *Arabidposis*[41,42], consistent with our observations of elevated expression of genes involved in flavonoid biosynthesis in the cacao somatic embryos relative to zygotic embryos. Flavonoids are commonly associated with plant stress responses and they appear to play a role in controlling growth and development in response to auxin and ethylene signaling by modulating auxin transport [42,43] and thus are critical to regulation of seed development. In summary, the observation that large numbers of genes involved in ethylene function were up-regulated in somatic embryos is consistent with the hypothesis that ethylene-mediated stress response plays a significant role in the abnormal development of cacao somatic embryos.

### Regulation of fatty acid biosynthesis in somatic embryos

We also observed high levels of mis-expression of genes encoding for key enzymes controlling lipid metabolism in somatic embryos. We observed increased expression of *FAB2*[44,45], *KASII*[46] and *FatA*[47] all involved in synthesis of oleic acid, a substrate for polyunsaturated fatty acid biosynthesis. Furthermore, the up-regulated expression of omega 3 and omega 6 desaturase genes *FAD2*[45] and *FAD7/8*[48] is also consistent with our recent observation that compared to cacao seeds, mature cacao SEs contain a higher proportion of polyunsaturated fatty acids, including linoleic acid and α-linolenic acid, but lower percentage of stearic acid and oleic acid (unpublished data, Yufan Zhang), which is characteristic of an immature zygotic seed and cacao leaves [31].

## Conclusions

A very large portion of the genome is differentially regulated during cacao zygotic embryo maturation, similar to what has been reported for other species. We conclude that the major gene expression programs during the maturation phase of zygotic embryogenesis are only partially expressed during somatic embryo maturation. This is not surprising considering the remarkably different environments and developmental pathways involved. Our results indicate that auxin, ethylene and flavonoid biosynthesis and regulation genes are abnormally expressed in SEs and these pathways could be very important targets for future optimization of the somatic embryogenesis process.

This research has provided a comprehensive list of differentially expressed genes and their expression profiles that could be further developed as markers for the maturation of ZE and SE. The data represents useful new knowledge into the metabolic processes associated with both of these embryo developmental processes in *T. cacao*, and provide a resource for functional genomics research and *in vitro* optimizations of medium and environmental conditions for improvement of the cacao SE system. These findings will provide hypothesis and tools to further dissect these factors and lead to improved somatic embryo protocols for cacao.

## Methods

### Plant materials

#### Zygotic embryos (ZE)

*Cacao* pods were obtained by hand pollination of a genotype Scavina6 (Sca6) plants with pollen from a genotype West African Amelonado plant in the greenhouse at The Pennsylvania State University. Developing fruits were harvested during maturation from 14 weeks after pollination (WAP), which corresponded to the Torpedo stage (T-ZE), 16 WAP (early-full, EF-ZE), 18 WAP (late-full, LF-ZE) to 20 WAP (mature, M-ZE), when pods are fully ripe. Zygotic embryos were extracted from each pod, cotyledon tissue was excised, frozen in liquid nitrogen and stored at −80°C for RNA extraction. For each developmental stage, four sets of biological replicates were frozen.

#### Somatic embryos (SE)

Secondary SEs were generated in temporary immersion system bioreactors [49,50] from flower parts of genotype Sca6 as previously described [18] with minor modifications as follows: glass beads (2 mm diameter) were used to support the embryos in the bioreactors. A detailed description of somatic embryo stages has been previously reported [18,19,51,52]. SEs at early torpedo developmental stage were selected and transferred to embryo development (ED) medium (20 embryos per bioreactor). Tissues were harvested after 2 weeks on ED medium at the following two developmental stages: 1) whole late torpedo SEs (LT-SE) cultured on 30 g/L sucrose and 2) cotyledon tissue from mature SEs (M-SE) cultured on 60 g/L sucrose (see Figure 1). One gram of tissue per biological replicate was frozen in liquid nitrogen and stored at −80°C for RNA extraction. Four biological replicates were collected for each developmental stage and immediately frozen in liquid nitrogen.

### Total RNA extraction

Total RNA was extracted using the method described by [53,54]. Briefly, frozen samples were ground in liquid nitrogen and the powder was suspended in extraction buffer containing 4 M guanidinium isothiocyanate, 0.24 M sodium acetate, 0.03 M N-lauroyl sarcosine sodium salt, 22.5 mM PVP-40 (MW 40,000) and 14 mM β-mercaptoethanol. After centrifugation at 14,000 g for 30 min at 4°C, RNAs were chloroform extracted, precipitated with isopropanol, washed with 70% ethanol and then re-suspended in RNase-free sterile water. The RNA was then treated with DNase l (Invitrogen) and integrity was assessed using a RNA 6000 Nano assay bioanalyzer (Aglient), RNA with a RNA integrity number of 7 or above was used.

### Microarray design and fabrication

Based on the genome sequence of *Theobroma cacao* (Criollo) [1], a set of 28,798 predicted gene models was used to extract all predicted coding sequences (CDS) and for those genes with a 3' untranslated region (UTR) sequences prediction, we extracted 22,489 additional 3’UTR sequences. The 3'UTRs were included to allow gene specific probes to resolve expression specificities of multi-gene families. The resulting 51,287 sequences were used to design unique 60-mer oligonucleotide probes (NimbleGen Chip Design Service). A final set of 134,357 60-mer oligonucleotide sequences were selected (84,650 CDS probes and 49,707 3’UTR probes) with an average of 4.67 probes per gene sequence (2.93 probes per CDS and 2.21 probes per 3’UTRs) with exception of 4,473 of target sequences that were either too short or of limited complexity so that no probes could be designed. Only 46 genes of the original 28,798 gene models were not represented on the array.

The individual probe elements were synthesized on NimbleGen 12-plex microarrays, which contains 135,000 probe locations on each of 12 sub-arrays per slide. Randomized control sequences were used to fill any remaining spaces on the array.

### Probe labeling, hybridization and detection

Hybridizations were performed by the Genomics Core Facility at PSU according to published facility protocols for NimbleGen microarrays [55]. One μg of total RNA (RNA Integrity Number (RIN) number of 7 or above) was amplified using mRNA amplification kit (Amino Allyl MessageAmp II™, Ambion, Austin, TX, AM1753) prior to labeling and hybridization. The aRNA was dye coupled with Cy3 or Cy5 (GE Health Care #RPN5661) and subsequently purified according to the Ambion Kit instructions. The 24 samples were paired and for one 12-plex array each Cy3 labeled sample (1.5 μg) was combined a Cy5 labeled sample (with 1.5 μg) and fragmented using RNA Fragmentation Reagents (Ambion AM8740) according the manufacturer’s instructions. Following fragmentation the samples were dried down completely in a speed-vac. The resuspended pairs of samples were subsequently hybridized to a single 12-plex array at 42°C for 18 hours. Arrays were washed according to manufacturer’s instructions (Roche NimbleGen) to remove non-specifically bound target and were scanned with an Axon 4000A scanner using associated software.

### Data analysis

The R programming environment [56] and Bioconductor software were used to perform the analysis [57]. Image plots of the probe-level data allowed for an assessment of microarray data anomalies. Log_2_ image plots for the 24 arrays indicated ‘normal’ high-quality array data and background and quality check were performed using the control probes.

After quality pre-processing, data was transformed to generate a final expression value for each gene. We used the LIMMA package to perform the background adjustment, normalization and summarization [58]. The RMA procedure, which performs a convolution background correction, quantile normalization and summarization based on a multi-array model fit robustly using the median polish algorithm, was used to obtain the average Log_2_ expression values for each gene (average of all probes) ranging from 2 to 16. Background noise was calculated as the average intensity levels of the control probes and a value of 6 (Log_2_) was used as a cutoff value to eliminate any signals due to noise. Statistical significance was using a moderated t-statistic implemented in the ‘LIMMA’ package in Bioconductor. It is based on an empirical Bayes approach detailed in [59]. The moderated t-statistics and their corresponding p-values were computed for statistical analysis. In this study, we performed this analysis with cut-off p < 0.01 and ≥ 2-fold change as our regular cutoff in statistical analysis. The data was deposited in NCBI's Gene Expression Omnibus (GEO) [60], GEO project GSE55476 http://www.ncbi.nlm.nih.gov/geo/query/acc.cgi?acc=GSE55476).

### Clustering analysis

Normalized and filtered data from the SE and ZE group were analyzed by LIMMA package in R. The differentially expressed genes were clustered according to their expression patterns across the 6 samples into different sets using the k-means unsupervised clustering technique. In brief, this algorithm arbitrarily separated the genes (“vectors”) into different groups. The centroid of each group was calculated by averaging the coordinates attached to each gene. In one iteration, each clone was then reassigned to the centroid to which it is closest and the coordinates of the centroids were recalculated.

### GO annotation using Blast2GO

All of the predicted genes in the cacao genome database [1] were functionally annotated using Blast2GO [61] with default parameters against the non-redundant (nr) protein sequence database. Similarly, Blast2GO software v2.5.0 was used to obtain Gene ontology (GO) information by GO mapping function from retrieved database matches. Finally, the ‘ANNEX (Annotation Argumentation)’ function was used to refine annotations. GOslim ‘goslim_plant.obo’ was used to generate specific GO terms. The output data was exported as annotation format files (annot) and the gene IDs in the different clusters were selected to generate the pie charts. The hierarchical representation of the GO was structured according to different levels, from the highest (level 1) parents corresponding to the 3 main GO categories (Cellular Component, Biological Progress, Molecular Function) to the lowest, more specialized child terms (level 2, 3, 4 etc.). In our study, GO annotated datasets were represented at level 2.

### GO enrichment

The whole genome GO categories for the differentially expressed genes were identified with Blast2GO software using GO enrichment analysis (Fisher’s Exact Test). For this analysis, the *cacao* annotation file (described above) was used as a reference to perform the analysis. GO terms with *p-values* less than 0.01 were considered to be significantly different and enriched in our sets of differentially expressed genes.

### Metabolic pathway analysis with MapMan

A MapMan [62] mapping file that included all predicted genes from *Theobroma cacao* genome (Criollo) database [1] was generated using the Mercator pipeline for automated sequence annotation via the MapMan website. In brief, a 28,802 peptide sequences FASTA file was uploaded to the Mercator tool (assigning default parameters plus conservative and InterProScan) for comparison to reference databases containing known protein sequences in order to get functional annotation. The generated mapping file contained Tc gene IDs with assigned MapMan bins (gene functional categories). This file was used for the MapMan analysis, which included the placement and visualization of the significant genes into metabolic pathways. Differentially expressed genes that were enriched from the prior microarray analysis were grouped in their respective functional categories. The fold differences in gene expression between M-SE and M-ZE were included to each gene. A positive value, shown in blue, represents an increase of gene expression in M-SE compared to M-ZE. A negative value, shown in red, represents a decrease of gene expression in M-SE compared to M-ZE.

### Quantitative real-time PCR analysis

Gene specific primers (10 randomly selected genes from the microarray and 2 control housekeeping genes were synthesized at the Penn State Nucleic Acid Facility with a MerMade12 automated DNA synthesizer (Bioautomation, Plano TX) (Additional file 18). Gene specific fluorescent probes were synthesized by Biosearch Technologies (Novato, CA). The fluorescent label used at the 5' end on the cacao genes probes was 6-carboxyfluorescein (6-FAM) and quencher at the 3' end of the gene probe was BHQ1 (Biosearch). PCR reactions in total volume of 25 μl included: 5 μl of cDNA (~12.5 ng), 12.5 μl 2X TaqMan® Universal Master Mix (#4304437, Applied Biosystems, Foster City, CA), 400 nmoles of each primer, and 200 nmoles of probe. The PCR reactions were run in 96 well thin-walled PCR plates in an Applied Biosystems 7300 Q-PCR system (Foster City, CA) with the following reaction conditions; 2 min at 50°C., 10 min at 95°C, followed by 40 cycles of 15 sec at 95°C and 1 min at 60°C. Each sample was amplified in duplicate and the results were averaged.

The mean expression of two cacao housekeeping control genes, actin and ubiquitin (*Tc05g027250* and *Tc09g021610*) was used to normalize the data. Amplification efficiency of all target and reference genes was calculated from the slopes of the dilution curves for each sample (E = 10^(−1/slope)^-1) [63]. Average efficiency for each gene was then calculated and used for efficiency data correction. Data normalization, efficiency correction, statistical randomization test and relative transgenic/control non-transgenic expression ratios were computed using REST software [64]. Ratios (fold difference) with *p-values* less than 0.05 were considered significant.

## Competing interests

The authors declare that they have no competing interests.

## Authors' contributions

SM contributed overall project coordination, experimental design, data analysis and interpretation; SF performed MapMan data analysis, transcription factor genes data analysis and interpretation, microarray data forming and submission to GEO database; XS performed microarray data analysis; NN performed the pollinations of the trees, zygotic embryo sample collection, somatic embryo tissue culture and sample collection, RNA extraction, qRT-PCR analsyis; YZ performed lipid biosynthesis genes data analysis and interpretation; WC and MG contributed to experimental design and data interpretation; All authors contributed to manuscript preparation and editing. All authors read and approved the final manuscript.

## Supplementary Material

Additional file 1 **Genes differentially expressed during zygotic embryo maturation.** Relative gene expression values (log_2_) of all genes up- or down-regulated during zygotic embryo maturation relative to torpedo stage zygotic embryos (T-ZE). List includes genes up- or down-regulated in at least one of the developmental stages compared and T-ZE (cut-off of *p* < 0.01 and ≥ 2-fold change). The fold difference of normalized expression values of the mature stage of zygotic embryos relative to expression values of torpedo embryos are indicated (Fold Difference M-ZE/T-ZE). Gene ID numbers and predicted function (Description) are indicated based on the criollo cacao genome browser V1.0 annotations [1]. Click here for file

Additional file 2 **List of GO categories enriched during somatic and zygotic embryo maturation in cacao.** GO enrichment analysis for zygotic embryo maturation (ZE) significant genes (Figure 2D); GO enrichment in Somatic embryo maturation (SE) significant genes compared to Torpedo-Zygotic Embryo stage T-ZE (Figure 3D); GO enrichment in SE significant genes compared to Mature-ZE (M-ZE, Figure 4D); GO enrichment in ZE and SE significant genes potentially involved in major metabolic pathways (Figure 5C). P-value of significance of enrichment compared to % representation in the whole genome is presented. The percentage of each GO category represented in the differentially expressed gene list and in the whole genome are indicated. For each GO enriched category, the percentage of significantly up or down regulated genes are also indicated. Click here for file

Additional file 3 **Differential gene expression between late torpedo and mature developmental stages of somatic embryogenesis.** Relative gene expression values (log_2_) of all genes up- or down-regulated comparing late torpedo (LT-SE) and mature (M-SE) stages of somatic embryogenesis (cut-off of *p* < 0.01 and ≥ 2-fold change). The fold difference of normalized expression values of the mature stage to the torpedo stage somatic embryos are indicated (Fold Difference M-SE/LT-SE). Gene ID numbers and predicted function (Description) are indicated based on the criollo cacao genome browser V1.0 annotations [1]. Click here for file

Additional file 4 **Cluster analysis of differentially expressed genes between late torpedo and mature developmental stages of somatic embryogenesis. (A)** Heatmap of all differentially expressed genes between late torpedo (LT-SE) and mature (M-SE) developmental stages of somatic embryogenesis (SE). Comparison of the two SE stages using K-means clustering of the transformed data identified 2213 up-regulated and 2207 down-regulated genes distributed in 4 clusters. **(B)** GO enrichment analysis (FDR < 0.01, Material and Methods) of all differentially expressed genes between LT-SE and M-SE. GO categories in red labels indicate genes enriched in SE but not in ZE developmental stages. Click here for file

Additional file 5 **Genes differentially expressed during somatic embryo maturation referenced to torpedo stage of zygotic embryogenesis.** Relative gene expression values (log_2_) of all genes up- or down-regulated comparing late torpedo eomatic embryos (LT-SE) and mature somatic embryos (M-SE) using torpedo zygotic embryo (T-ZE) as a reference. Using a differential expression cut-off of *p* < 0.01 and ≥ 2-fold change filter, we identified 10,637 differentially expressed genes in. The fold difference of normalized expression values of the mature stage somatic embryos and the torpedo zygotic embryo are indicated (Fold Difference M-SE/T-ZE). Gene ID numbers and predicted function (Description) are indicated based on the criollo cacao genome browser V1.0 annotations [1]. Click here for file

Additional file 6 **Genes differentially expressed during somatic embryo maturation referenced to mature stage of zygotic embryogenesis.** Relative gene expression values (log_2_) of all genes up- or down-regulated comparing late torpedo somatic embryos (LT-SE) and mature somatic embryos (M-SE) using mature zygotic embryo (M-ZE) as a reference (cut-off of *p* < 0.01 and ≥ 2-fold change). The fold difference of normalized expression values of the mature stage somatic embryos and the mature zygotic embryo are indicated (Fold Difference M-SE/M-ZE). Gene ID numbers and predicted function (Description) are indicated based on the criollo cacao genome browser V1.0 annotations [1]. Click here for file

Additional file 7 **Patterns of gene expression during zygotic embryo maturation.** Differentially expressed genes across the 4 developmental stages of zygotic embryo maturation were grouped into 10 clusters using the K-means clustering algorithm: torpedo (T), early-full (EF), late-full (LF), mature (M). The 10 ZE clusters identified include: up-regulated genes (clusters III, IV and VI), down-regulated genes (clusters I, II and VII), up-down-regulated (clusters V and VIII) and down-up-regulated (clusters IX and X). The expression values (log_2_) relative to the T-ZE stage are represented on the y-axis and the developmental stage on the x-axis. The red lines represent the mean of the expression values in each cluster. Pie chart GO classifications for biological process (P) are represented. The genes included in this analysis were those associated level 2 biological process functional GO term annotations: 5700 genes in the ZE group (55.4% of the 10,288) and 5754 genes in the SE group (56.3% of the 10, 210). Unclassified genes were omitted from the pie charts; numbers of classified genes are shown in brackets under the chart. The percentages of GO terms enriched in each cluster are indicated. Click here for file

Additional file 8 **Patterns of gene expression during somatic embryo maturation referenced to torpedo stage of zygotic embryogenesis.** Differentially expressed genes between 2 developmental stages of SE using to T-ZE as reference were grouped into 5 clusters each using the K-means clustering algorithm. Expression patterns were sorted into three general classes: genes with higher expression in SE (clusters I and III), down-regulated in T-SE (clusters IV) and up-regulated in T-SE (clusters II and V). The relative expression values (log_2_) are represented on the y-axis and embryo developmental stages on the x-axis. The red line depicts the mean expression values in each cluster. Pie chart classifications for biological process (P) are represented under the clusters. The genes included in this analysis were those associated level 2 biological process functional GO term annotations: 5700 genes in the ZE group (55.4% of the 10,288) and 5754 genes in the SE group (56.3% of the 10, 210). Unclassified genes were omitted from the pie charts, numbers of classified genes are shown in brackets under the chart. The percentages of GO terms enriched in each of the whole sub-clusters are indicated. Click here for file

Additional file 9 **Transcription factor genes significantly regulated during cacao zygotic embryo maturation.** Relative gene expression values (log_2_) of Transcription factor genes (TF) genes up- or down-regulated during zygotic embryo (ZE) maturation compared to torpedo stage of zygotic embryos (T-ZE). A differential expression cutoff of *p* < 0.01 and ≥ 2-fold change filter was used. The fold difference of normalized expression values between mature stage zygotic embryos and torpedo zygotic embryo are indicated (Fold Difference M-ZE/T-ZE). Gene ID numbers and predicted function (Description) are indicated based on the criollo cacao genome browser V1.0 annotations [1]. Click here for file

Additional file 10 **Transcription factor genes significantly regulated during cacao somatic embryo maturation referenced to mature zygotic embryos.** Relative gene expression values (log_2_) of TF genes up- or down-regulated in late torpedo-SE (LT-SE) and mature-SE (M-SE) referenced to mature-ZE (M-ZE). A differential expression cutoff of *p* < 0.01 and ≥ 2-fold change filter was used. The fold difference of normalized expression values between mature stage zygotic embryos and torpedo zygotic embryo are indicated (Fold Difference M-SE/M-ZE). Gene ID numbers and predicted function (Description) are indicated based on the criollo cacao genome browser V1.0 annotations [1]. Click here for file

Additional file 11 **Overview of the function of differentially expressed transcription factor genes comparing mature somatic to mature zygotic embryo stages.** Diagram generated by MapMan Software using *Theobroma cacao* 28 K gene ontology mapping file. Color coded bars represent the ratio of gene expression in somatic vs zygotic embryogenesis (blue = genes more highly expressed in zygotic embryos, red = genes expressed more highly in somatic embryos). Using the overview pathway tool (not shown) the genes were grouped into the following bins: RNA - regulation (290 genes), Development (32 genes), protein synthesis/degradation (34 genes), Hormone metabolism (18 genes), DNA synthesis (6), Signaling (3), Cell organization (9 genes), Miscellaneous (3 genes), Calvin cycle (1 gene), Abiotic stress heat (1 gene), Not assigned (31 genes). Two of the categories identified of particular interest to this study were hormone regulation and cell development, accounting for 35 and 32 genes respectively. Click here for file

Additional file 12 **Lipid biosynthesis genes differentially regulated during cacao somatic embryo maturation referenced to mature zygotic embryos.** Relative gene expression values (log_2_) of lipid biosynthesis genes up- or down-regulated in late torpedo somatic embryos (LT-SE) and mature somatic embryos (M-SE) referenced to mature zygotic embryos (M-ZE). A differential expression cutoff of *p* < 0.01 and ≥ 2-fold change filter was used. The fold difference of normalized expression values between mature stage zygotic embryos and torpedo zygotic embryo are indicated (Fold Difference M-SE/M-ZE). Gene ID numbers and predicted function (Description) are indicated based on the criollo cacao genome browser V1.0 annotations [1]. Click here for file

Additional file 13 **Flavonoid biosynthesis genes differentially regulated during cacao zygotic embryo maturation referenced to torpedo developmental stage.** Relative gene expression values (log_2_) of flavonoid biosynthesis genes up- or down-regulated in late torpedo somatic embryos (LT-SE) and mature somatic embryos (M-SE) referenced to torpedo zygotic embryos (T-ZE). A differential expression cutoff of *p* < 0.01 and ≥ 2-fold change filter was used. The fold difference of normalized expression values between mature stage zygotic embryos and torpedo zygotic embryo are indicated (Fold Difference M-SE/M-ZE). Gene ID numbers and predicted function (Description) are indicated based on the criollo cacao genome browser V1.0 annotations [1]. Click here for file

Additional file 14 **Flavonoid biosynthesis genes differentially regulated during cacao somatic embryo maturation referenced to mature zygotic embryos.** Relative gene expression values (log_2_) of flavonoid biosynthesis genes up- or down-regulated in late torpedo somatic embryos (LT-SE) and mature somatic embryos (M-SE) referenced to mature zygotic embryos (M-ZE). A differential expression cutoff of *p* < 0.01 and ≥ 2-fold change filter was used. The fold difference of normalized expression values between mature stage zygotic embryos and torpedo zygotic embryo are indicated (Fold Difference M-SE/M-ZE). Gene ID numbers and predicted function (Description) are indicated based on the criollo cacao genome browser V1.0 annotations [1]. Click here for file

Additional file 15 **Overview of the function of differentially expressed genes encoding enzymes in various secondary metabolite biosynthetic pathways.** MapMan diagram was generated using the *Theobroma cacao* 28 K gene ontology mapping file highlighting the enrichment of three groups: flavonoids, phenylpropanoids and lignin. Blue represents genes that are expressed higher in mature somatic embryos (M-SE) compared to mature zygotic embryos (M-ZE) while red represent a decrease in expression levels. Click here for file

Additional file 16 **Seed storage protein genes significantly regulated during cacao zygotic and somatic embryo maturation.** A) Significantly regulated seed storage protein genes during zygotic embryo maturation using torpedo zygotic embryos (T-ZE) as reference. B) Significantly regulated seed storage protein genes during somatic embryo maturation using mature zygotic embryos (M-ZE) as reference. Relative gene expression values (log_2_) are presented, cutoff set at *p* < 0.01 and ≥ 2-fold change. The fold difference of normalized expression values between mature stage zygotic embryos and torpedo zygotic embryo are indicated. Gene ID numbers and predicted function (Description) are indicated based on the criollo cacao genome browser V1.0 annotations [1]. Click here for file

Additional file 17 **Verification by qRT-PCR of microarray results of 10 differentially expressed genes.** The ratio of expression values (M-ZE/LT-SE) of ten genes obtained by qRT-PCR (red bars) and microarray analysis (blue bars). Click here for file

Additional file 18 Primer and probe sequences for TaqMan® assays of genes analyzed by QPCR. Click here for file

## References

[B1] Argout X Salse J Aury JM Guiltinan MJ Droc G Gouzy J Allegre M Chaparro C Legavre T Maximova S Abrouk M Murat F Fouet O Poulain J Ruiz M Roguet Y Rodier-Goud M Barbosa-Neto J Sabot F Kudrna D Ammiraju J Schuster S Carlson J Sallet E Schiex T Dievart A Kramer M Gelley L Shi Z Bérard A The genome of Theobroma cacao Nature Genetics 2011 43 2 101 108 21186351 10.1038/ng.736

[B2] Jenik PD Gillmor CS Lukowitz W Embryonic patterning in Arabidopsis thaliana Annu Rev Cell Dev Biol 2007 23 207 236 17539754 10.1146/annurev.cellbio.22.011105.102609

[B3] Linkies A Graeber K Knight C Leubner-Metzger G The evolution of seeds New Phytol 2010 186 4 817 831 20406407 10.1111/j.1469-8137.2010.03249.x

[B4] Esau K Embryo and Seedling Anatomy of Seed Plants 1977 2 New York: John Wiley & Sons 475 500

[B5] Braybrook SA Harada JJ LECs go crazy in embryo development Trends Plant Sci 2008 13 12 624 630 19010711 10.1016/j.tplants.2008.09.008

[B6] Spencer MW Casson SA Lindsey K Transcriptional profiling of the Arabidopsis embryo Plant Physiol 2007 143 2 924 940 17189330 10.1104/pp.106.087668 PMC1803724

[B7] Naik SK Chand PK Tissue culture-mediated biotechnological intervention in pomegranate: a review Plant Cell Rep 2011 30 5 707 721 21161233 10.1007/s00299-010-0969-7

[B8] Steward FC Pollard JK Patchett AA Witkop B The effects of selected nitrogen compounds on the growth of plant tissue cultures Biochimica et Biophysica Acta 1958 28 2 308 317 13535727 10.1016/0006-3002(58)90477-3

[B9] Zimmerman JL Somatic embryogenesis: A model for early development in higher plants Plant Cell 1993 5 10 1411 1423 12271037 10.1105/tpc.5.10.1411 PMC160372

[B10] Santos-Mendoza M Dubreucq B Baud S Parcy F Caboche M Lepiniec L Deciphering gene regulatory networks that control seed development and maturation in Arabidopsis Plant J 2008 54 4 608 620 18476867 10.1111/j.1365-313X.2008.03461.x

[B11] Broun P Transcriptional control of flavonoid biosynthesis: a complex network of conserved regulators involved in multiple aspects of differentiation in Arabidopsis Curr Opin Plant Biol 2005 8 3 272 279 15860424 10.1016/j.pbi.2005.03.006

[B12] Li HC Chuang K Henderson JT Rider SD Jr Bai Y Zhang H Fountain M Gerber J Ogas J PICKLE acts during germination to repress expression of embryonic traits Plant J 2005 44 6 1010 1022 16359393 10.1111/j.1365-313X.2005.02602.x PMC2488385

[B13] Cernac A Benning C WRINKLED1 encodes an AP2/EREB domain protein involved in the control of storage compound biosynthesis in Arabidopsis Plant J 2004 40 4 575 585 15500472 10.1111/j.1365-313X.2004.02235.x

[B14] Goldberg RB de Paiva G Yadegari R Plant embryogenesis: zygote to seed Science 1994 266 5185 605 614 17793455 10.1126/science.266.5185.605

[B15] Lopes MA Larkins BA Endosperm origin, development, and function Plant Cell 1993 5 10 1383 1399 8281040 10.1105/tpc.5.10.1383 PMC160370

[B16] Sabelli PA Larkins BA The development of endosperm in grasses Plant Physiology 2009 149 1 14 26 19126691 10.1104/pp.108.129437 PMC2613697

[B17] Berger F Grini PE Schnittger A Endosperm: an integrator of seed growth and development Curr Opin Plant Biol 2006 9 6 664 670 17011228 10.1016/j.pbi.2006.09.015

[B18] Maximova SN Alemanno L Young A Ferriere N Traore A Guiltinan MJ Efficiency, genotypic variability, and cellular origin of primary and secondary somatic embryogenesis of Theobroma cacao L Vitro Cellular & Developmental Biology - Plant 2002 2002 38 252 259

[B19] Li Z Traore A Maximova S Guiltinan MJ Somatic embryogenesis and plant regeneration from floral explants of cacao (*Theobroma cacao L.*) using thidiazuron Vitro Cell Dev Biol Plant 1998 34 293 299

[B20] Alemanno L Berthouly M Michaux-Ferriere N Somatic embryogenesis of cocoa from floral parts Plantations, Recherche, Developpement 1996 3 225 237

[B21] Alemanno L Berthouly M Michaux-Ferriere N A comparison between Theobroma cacao L. zygotic embryogenesis and somatic embryogenesis from floral explants Vitro Cellular & Developmental Biology - Plant 1997 33 3 163 172

[B22] Lopez-Baez O Bollon H Eskes A Petiard V Embryogenèse somatique de cacaoyer Theobroma cacao L. á partir de pièces florales Comptes Rendus de l'Académie des Sciences Paris 1993 316 579 584

[B23] Maximova SN Young A Pishak S Guiltinan MJ Field performance of Theobroma cacao L. plants propagated via somatic embryogenesis Vitro Cellular & Developmental Biology - Plant 2008 44 6 487 493

[B24] Traore A Somatic Embryogenesis, Embryo Conversion, Micropropagation and Factors Affecting Genetic Transformation of Theobroma Cacao L PhD thesis 2000 University Park: The Pennsylvania State University

[B25] Mitsuda N Ohme-Takagi M Functional analysis of transcription factors in Arabidopsis Plant Cell Physiol 2009 50 7 1232 1248 19478073 10.1093/pcp/pcp075 PMC2709548

[B26] Weber H Hellmann H Arabidopsis thaliana BTB/ POZ-MATH proteins interact with members of the ERF/AP2 transcription factor family The FEBS journal 2009 276 22 6624 6635 19843165 10.1111/j.1742-4658.2009.07373.x

[B27] Zheng Q Zheng Y Perry SE AGAMOUS-Like15 promotes somatic embryogenesis in Arabidopsis and soybean in part by the control of ethylene biosynthesis and response Plant Physiol 2013 161 4 2113 2127 23457229 10.1104/pp.113.216275 PMC3613480

[B28] Boutilier K Offringa R Sharma VK Kieft H Ouellet T Zhang L Hattori J Liu CM van Lammeren AA Miki BL Custers JB van Lookeren Campagne MM Ectopic expression of BABY BOOM triggers a conversion from vegetative to embryonic growth Plant Cell 2002 14 8 1737 1749 12172019 10.1105/tpc.001941 PMC151462

[B29] Nambara E Keith K Mccourt P Naito S A regulatory role for the ABI3 Gene in the establishment of embryo maturation in *Arabidopsis thaliana* Development 1995 121 3 629 636

[B30] Finkelstein RR Lynch TJ The Arabidopsis abscisic acid response gene ABI5 encodes a basic leucine zipper transcription factor Plant Cell 2000 12 4 599 609 10760247 10.1105/tpc.12.4.599 PMC139856

[B31] Patel VK Shanklin J Furtek DB Changes in fatty-acid composition and stearoyl-acyl carrier protein desaturase expression in developing *Theobroma-cacao* L embryos Planta 1994 193 1 83 88

[B32] Lepiniec L Debeaujon I Routaboul JM Baudry A Pourcel L Nesi N Caboche M Genetics and biochemistry of seed flavonoids Annual Review of Plant Biology 2006 57 405 430 10.1146/annurev.arplant.57.032905.105252 16669768

[B33] Brown DE Rashotte AM Murphy AS Normanly J Tague BW Peer WA Taiz L Muday GK Flavonoids act as negative regulators of auxin transport in vivo in arabidopsis Plant Physiol 2001 126 2 524 535 11402184 10.1104/pp.126.2.524 PMC111146

[B34] Lazar G Goodman HM MAX1, a regulator of the flavonoid pathway, controls vegetative axillary bud outgrowth in Arabidopsis Proceedings of the National Academy of Sciences USA 2006 103 2 472 476 10.1073/pnas.0509463102 PMC1324789 16387852

[B35] Gutierrez L Van Wuytswinkel O Castelain M Bellini C Combined networks regulating seed maturation Trends in Plant Science 2007 12 7 294 300 17588801 10.1016/j.tplants.2007.06.003

[B36] Itoh Y Kitamura Y Arahira M Fukazawa C Cis-acting regulatory regions of the soybean seed storage 11S globulin gene and their interactions with seed embryo factors Plant Mol Biol 1993 21 6 973 984 8490143 10.1007/BF00023596

[B37] Higashi Y Hirai MY Fujiwara T Naito S Noji M Saito K Proteomic and transcriptomic analysis of Arabidopsis seeds: molecular evidence for successive processing of seed proteins and its implication in the stress response to sulfur nutrition Plant J 2006 48 4 557 571 17059406 10.1111/j.1365-313X.2006.02900.x

[B38] Gliwicka M Nowak K Balazadeh S Mueller-Roeber B Gaj MD Extensive Modulation of the Transcription Factor Transcriptome during Somatic Embryogenesis in Arabidopsis thaliana PLoS ONE 2013 8 7 1 20 10.1371/journal.pone.0069261 PMC3714258 23874927

[B39] Kong L Yeung E Effects of ethylene and ethylene inhibitors on white spruce somatic embryo maturation Plant Sci 1994 104 1 71 80

[B40] El Meskaoui A Tremblay FM Involvement of ethylene in the maturation of black spruce embryogenic cell lines with different maturation capacities Journal of Experimental Botany 2001 52 357 761 769 11413212 10.1093/jexbot/52.357.761

[B41] Buer CS Sukumar P Muday GK Ethylene modulates flavonoid accumulation and gravitropic responses in roots of Arabidopsis Plant Physiol 2006 140 4 1384 1396 16489132 10.1104/pp.105.075671 PMC1435817

[B42] Lewis DR Ramirez MV Miller ND Vallabhaneni P Ray WK Helm RF Winkel BS Muday GK Auxin and ethylene induce flavonol accumulation through distinct transcriptional networks Plant Physiol 2011 156 1 144 164 21427279 10.1104/pp.111.172502 PMC3091047

[B43] Grunewald W De Smet I Lewis DR Lofke C Jansen L Goeminne G Vanden Bossche R Karimi M De Rybel B Vanholme B Teichmann T Boerjan W Van Montagu MCE Gheysenc G Mudaye GK Friml J Beeckman T Transcription factor WRKY23 assists auxin distribution patterns during Arabidopsis root development through local control on flavonol biosynthesis Proc Natl Acad Sci U S A 2012 109 5 1554 1559 22307611 10.1073/pnas.1121134109 PMC3277162

[B44] Lightner J Wu J Browse J A mutant of Arabidopsis with increased levels of stearic acid Plant Physiol 1994 106 4 1443 1451 12232421 10.1104/pp.106.4.1443 PMC159684

[B45] Okuley J Lightner J Feldmann K Yadav N Lark E Browse J Arabidopsis FAD2 gene encodes the enzyme that is essential for polyunsaturated lipid synthesis Plant Cell 1994 6 1 147 158 7907506 10.1105/tpc.6.1.147 PMC160423

[B46] Pidkowich MS Nguyen HT Heilmann I Ischebeck T Shanklin J Modulating seed β-ketoacyl-acyl carrier protein synthase II level converts the composition of a temperate seed oil to that of a palm-like tropical oil Proc Natl Acad Sci U S A 2007 104 11 4742 4747 17360594 10.1073/pnas.0611141104 PMC1838670

[B47] DeVeaux LC Cronan JE Jr Smith TL Genetic and biochemical characterization of a mutation (fatA) that allows trans unsaturated fatty acids to replace the essential cis unsaturated fatty acids of Escherichia coli J Bacteriol 1989 171 3 1562 1568 2646287 10.1128/jb.171.3.1562-1568.1989 PMC209781

[B48] Collados R Andreu V Picorel R Alfonso M A light-sensitive mechanism differently regulates transcription and transcript stability of omega3 fatty-acid desaturases (FAD3, FAD7 and FAD8) in soybean photosynthetic cell suspensions FEBS Letters 2006 580 20 4934 4940 16930600 10.1016/j.febslet.2006.07.087

[B49] Niemenak N Saare-Surminski K Rohsius C Ndoumou DO Lieberei R Regeneration of somatic embryos in Theobroma cacao L. in temporary immersion bioreactor and analyses of free amino acids in different tissues Plant Cell Rep 2008 27 4 667 676 18193427 10.1007/s00299-007-0497-2

[B50] Shaw S An Improved Temporary Immersion Bioreactor Design for Plant Tissue Culture Propagation Honors Thesis 2012 Pennsylvania State University: Univeristy Park

[B51] Traore A Guiltinan M Effects of carbon source and explant type on somatic embryogenesis of four cacao genotypes HortScience 2006 41 3 753 758

[B52] Maximova SN Young A Pishak S Miller C Traore A Guiltinan MJ Jain SM, Gupta PK Integrated system for propagation of *Theobroma cacao* L Protocol for Somatic Embryogenesis in Woody Plants 2005 Dordrecht, The Netherlands: Springer 209 229

[B53] Alemanno L Devic M Niemenak N Sanier C Guilleminot J Rio M Verdeil JL Montoro P Characterization of leafy cotyledon1-like during embryogenesis in *Theobroma cacao L* Planta 2008 227 4 853 866 18094994 10.1007/s00425-007-0662-4

[B54] Rodriguez Lopez CM Wetten AC Wilkinson MJ Progressive erosion of genetic and epigenetic variation in callus-derived cocoa (*Theobroma cacao*) plants New Phytol 2010 186 4 856 868 20406408 10.1111/j.1469-8137.2010.03242.x

[B55] Protocols for NimbleGen 4-plex and 12-plex microarrays http://www.huck.psu.edu/facilities/genomics-up/protocols/nimblegen-protocols

[B56] The R Project for Statistical Computing http://www.r-project.org/

[B57] Gentleman RC Carey VJ Bates DM Bolstad B Dettling M Dudoit S Ellis B Gautier L Ge Y Gentry J Hornik K Hothorn T Huber W Iacus S Irizarry R Leisch F Li C Maechler M Rossini AJ Sawitzki G Smith C Smyth G Tierney L Yang JY Zhang J Bioconductor: open software development for computational biology and bioinformatics Genome Biology 2004 5 10 R80 15461798 10.1186/gb-2004-5-10-r80 PMC545600

[B58] Ritchie ME Silver J Oshlack A Holmes M Diyagama D Holloway A Smyth GK A comparison of background correction methods for two-colour microarrays Bioinformatics 2007 23 20 2700 2707 17720982 10.1093/bioinformatics/btm412

[B59] Smyth GK Linear models and empirical bayes methods for assessing differential expression in microarray experiments Stat Appl Genet Mol Biol 2004 3 Article3 10.2202/1544-6115.1027 16646809

[B60] Edgar R Domrachev M Lash AE Gene Expression Omnibus: NCBI gene expression and hybridization array data repository Nucleic Acids Res 2002 30 1 207 210 11752295 10.1093/nar/30.1.207 PMC99122

[B61] Conesa A Gotz S Garcia-Gomez JM Terol J Talon M Robles M Blast2GO: a universal tool for annotation, visualization and analysis in functional genomics research Bioinformatics 2005 21 18 3674 3676 16081474 10.1093/bioinformatics/bti610

[B62] Thimm O Bläsing O Gibon Y Nagel A Meyer S Krüger P Selbig J Müller LA Rhee SY Stitt M Mapman: a user-driven tool to display genomics data sets onto diagrams of metabolic pathways and other biological processes Plant J 2004 37 6 914 939 14996223 10.1111/j.1365-313x.2004.02016.x

[B63] Bustin SA A-Z of Quantitative PCR 2004 La Jolla, California: International University Line

[B64] Pfaffl MW Horgan GW Dempfle L Relative expression software tool (REST) for group-wise comparison and statistical analysis of relative expression results in real-time PCR Nucleic Acids Res 2002 30 9 e36 11972351 10.1093/nar/30.9.e36 PMC113859

