# Supplementary figures and images for: Genome-wide analysis reveals divergent patterns of gene expression during zygotic and somatic embryo maturation of Theobroma cacao L., the chocolate tree
Source: BMC Plant Biol. 2014 Jul 16;14:185. doi: 10.1186/1471-2229-14-185 (PMC4110631; doi:10.1186/1471-2229-14-185)

# Additional file 4

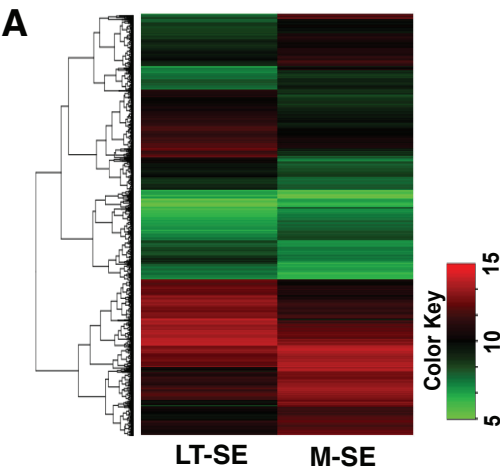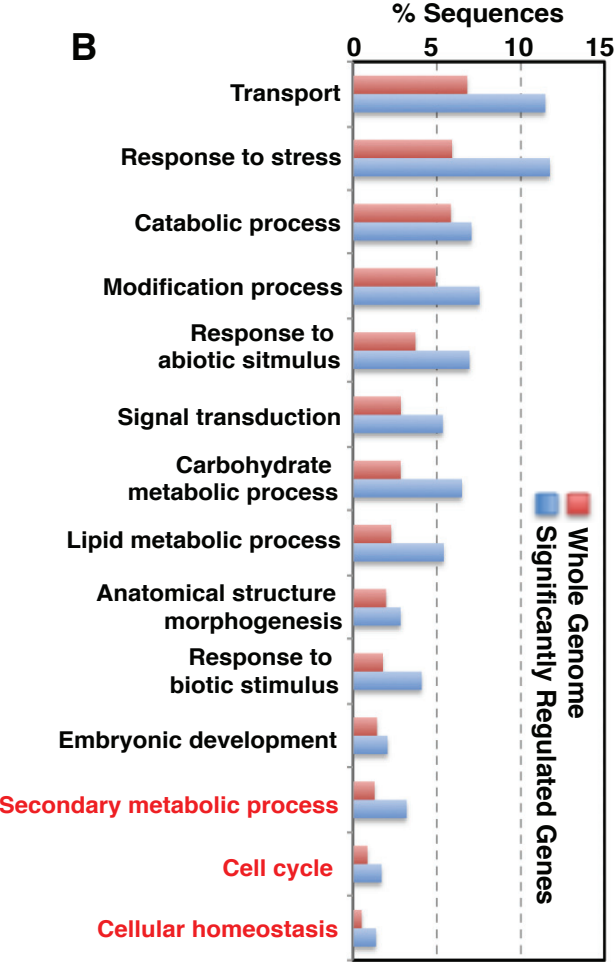

Supplement: Additional file 4 — Cluster analysis of differentially expressed genes between late torpedo and mature developmental stages of somatic embryogenesis. (A) Heatmap of all differentially expressed genes between late torpedo (LT-SE) and mature (M-SE) developmental stages of somatic embryogenesis (SE). Comparison of the two SE stages using K-means clustering of the transformed data identified 2213 up-regulated and 2207 down-regulated genes distributed in 4 clusters. (B) GO enrichment analysis (FDR < 0.01, Material and Methods) of all differentially expressed genes between LT-SE and M-SE. GO categories in red labels indicate genes enriched in SE but not in ZE developmental stages. [file 1471-2229-14-185-S4.pdf]

# Additional file 7

## Patterns of gene expression during zygotic embryo maturation

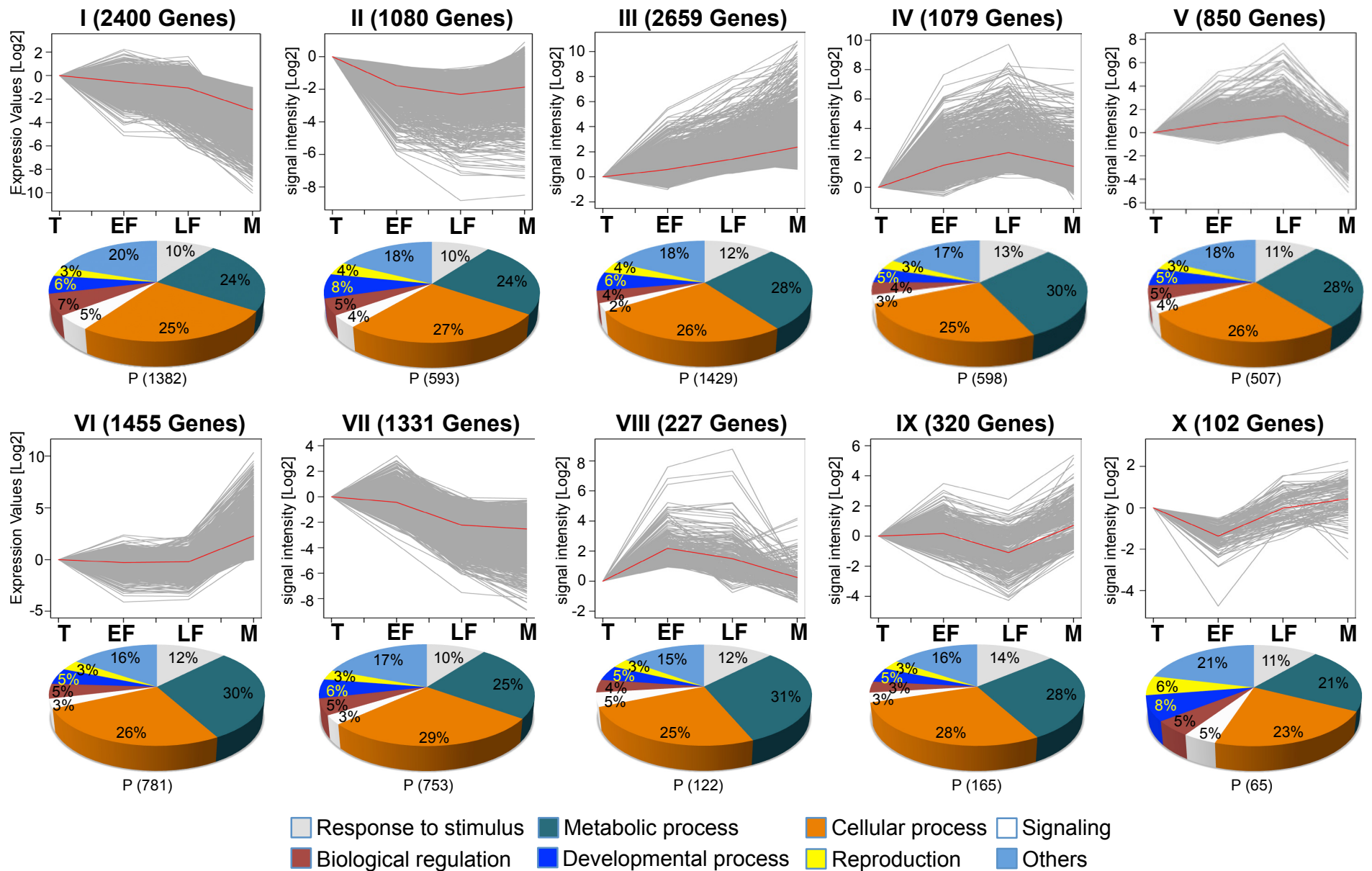

Supplement: Additional file 7 — Patterns of gene expression during zygotic embryo maturation. Differentially expressed genes across the 4 developmental stages of zygotic embryo maturation were grouped into 10 clusters using the K-means clustering algorithm: torpedo (T), early-full (EF), late-full (LF), mature (M). The 10 ZE clusters identified include: up-regulated genes (clusters III, IV and VI), down-regulated genes (clusters I, II and VII), up-down-regulated (clusters V and VIII) and down-up-regulated (clusters IX and X). The expression values (log2) relative to the T-ZE stage are represented on the y-axis and the developmental stage on the x-axis. The red lines represent the mean of the expression values in each cluster. Pie chart GO classifications for biological process (P) are represented. The genes included in this analysis were those associated level 2 biological process functional GO term annotations: 5700 genes in the ZE group (55.4% of the 10,288) and 5754 genes in the SE group (56.3% of the 10, 210). Unclassified genes were omitted from the pie charts; numbers of classified genes are shown in brackets under the chart. The percentages of GO terms enriched in each cluster are indicated. [file 1471-2229-14-185-S7.pdf]

## Additional file 8

### Patterns of gene expression during somatic embryo maturation

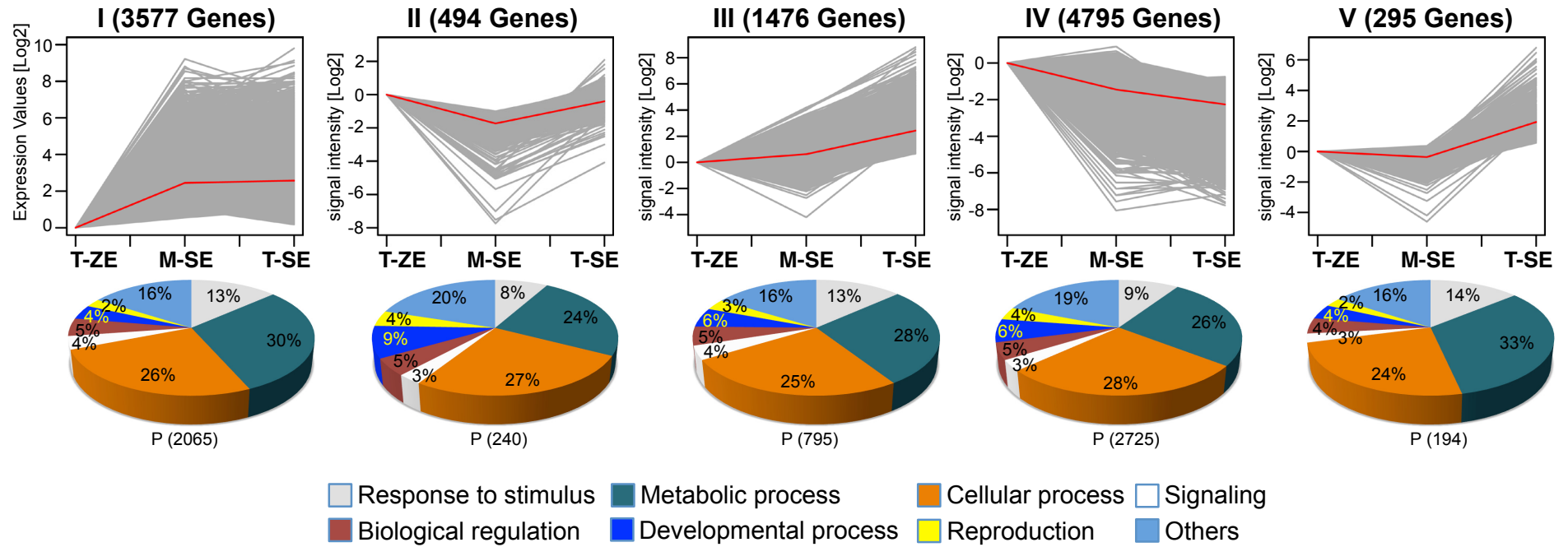

Supplement: Additional file 8 — Patterns of gene expression during somatic embryo maturation referenced to torpedo stage of zygotic embryogenesis. Differentially expressed genes between 2 developmental stages of SE using to T-ZE as reference were grouped into 5 clusters each using the K-means clustering algorithm. Expression patterns were sorted into three general classes: genes with higher expression in SE (clusters I and III), down-regulated in T-SE (clusters IV) and up-regulated in T-SE (clusters II and V). The relative expression values (log2) are represented on the y-axis and embryo developmental stages on the x-axis. The red line depicts the mean expression values in each cluster. Pie chart classifications for biological process (P) are represented under the clusters. The genes included in this analysis were those associated level 2 biological process functional GO term annotations: 5700 genes in the ZE group (55.4% of the 10,288) and 5754 genes in the SE group (56.3% of the 10, 210). Unclassified genes were omitted from the pie charts, numbers of classified genes are shown in brackets under the chart. The percentages of GO terms enriched in each of the whole sub-clusters are indicated. [file 1471-2229-14-185-S8.pdf]

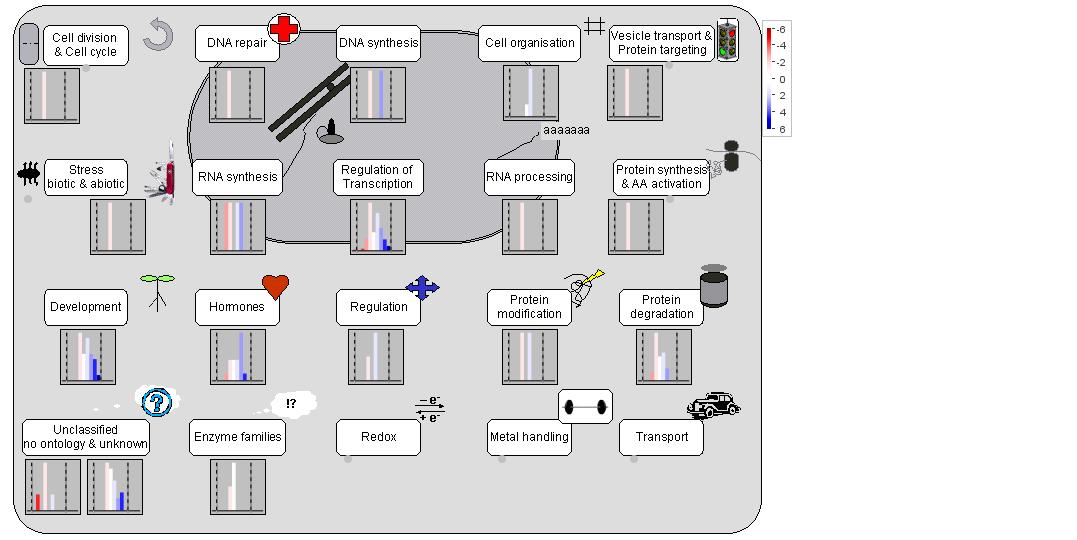

Supplement: Additional file 11 — Overview of the function of differentially expressed transcription factor genes comparing mature somatic to mature zygotic embryo stages. Diagram generated by MapMan Software using Theobroma cacao 28 K gene ontology mapping file. Color coded bars represent the ratio of gene expression in somatic vs zygotic embryogenesis (blue = genes more highly expressed in zygotic embryos, red = genes expressed more highly in somatic embryos). Using the overview pathway tool (not shown) the genes were grouped into the following bins: RNA - regulation (290 genes), Development (32 genes), protein synthesis/degradation (34 genes), Hormone metabolism (18 genes), DNA synthesis (6), Signaling (3), Cell organization (9 genes), Miscellaneous (3 genes), Calvin cycle (1 gene), Abiotic stress heat (1 gene), Not assigned (31 genes). Two of the categories identified of particular interest to this study were hormone regulation and cell development, accounting for 35 and 32 genes respectively. [file 1471-2229-14-185-S11.jpeg]

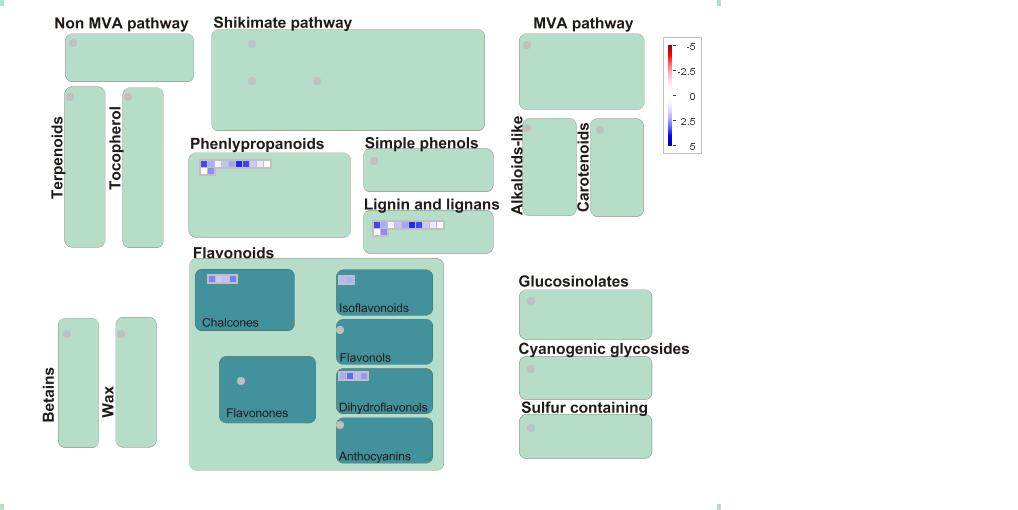

Supplement: Additional file 15 — Overview of the function of differentially expressed genes encoding enzymes in various secondary metabolite biosynthetic pathways. MapMan diagram was generated using the Theobroma cacao 28 K gene ontology mapping file highlighting the enrichment of three groups: flavonoids, phenylpropanoids and lignin. Blue represents genes that are expressed higher in mature somatic embryos (M-SE) compared to mature zygotic embryos (M-ZE) while red represent a decrease in expression levels. [file 1471-2229-14-185-S15.jpeg]

Additional file 17

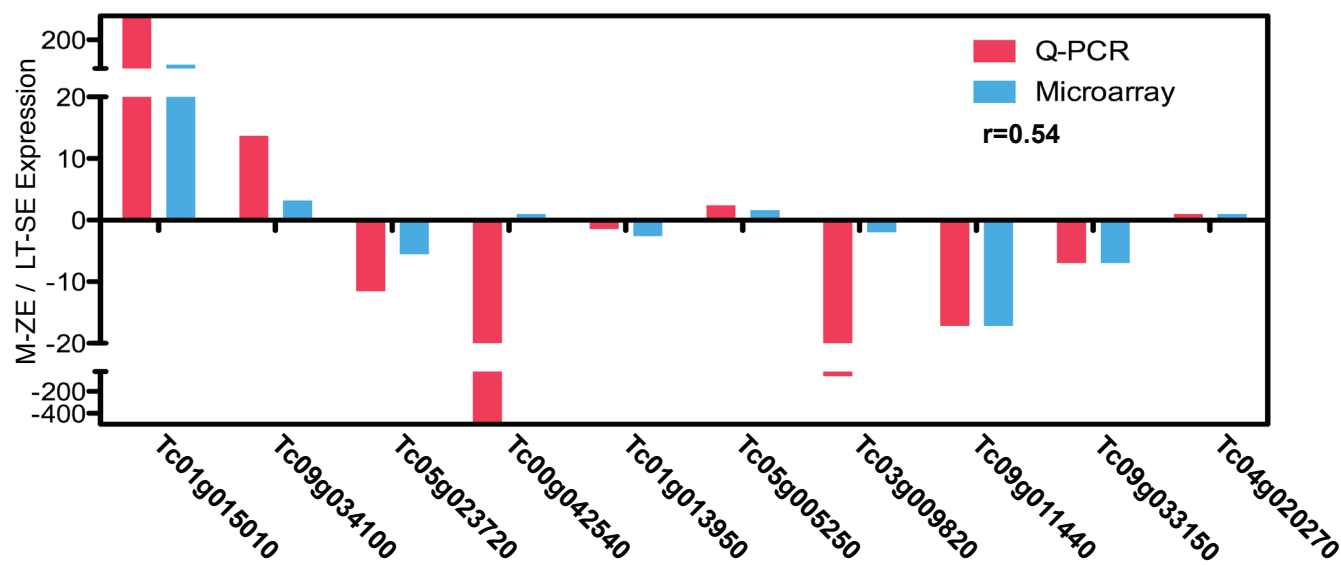

Supplement: Additional file 17 — Verification by qRT-PCR of microarray results of 10 differentially expressed genes. The ratio of expression values (M-ZE/LT-SE) of ten genes obtained by qRT-PCR (red bars) and microarray analysis (blue bars). [file 1471-2229-14-185-S17.pdf]
