# Supplementary material for: Genome-wide analysis reveals divergent patterns of gene expression during zygotic and somatic embryo maturation of Theobroma cacao L., the chocolate tree
Source: BMC Plant Biol. 2014 Jul 16;14:185. doi: 10.1186/1471-2229-14-185 (PMC4110631; doi:10.1186/1471-2229-14-185)
Supplement: Additional file 18 — Primer and probe sequences for TaqMan® assays of genes analyzed by QPCR. [file 1471-2229-14-185-S18.doc]

**Additional File 18. Primer and probe sequences for TaqMan® Q-PCR assays.**

| Sequence ID | Primer Sequences | Probe Sequences |
| --- | --- | --- |
| Tc01g015010 | 5'-TTCCTTTCTGCTTCATTACTACACAAC-3'  5'-TGAGCATCTTAGAAACACCTGC-3' | 5'-CAATGGTGCTTGTCAGCGGCA AGAA-3' |
| Tc09g034100 | 5'-CTCATTGCAGTGGTATTGGTTTTT-3'  5'-CGATGAGCATGAAGCTGCA-3' | 5'-ACCAGACAGACACGCGGAAGG CC-3' |
| Tc05g023720 | 5'-CGTCTCCCC GATATATACCTCTACTA-3'  5'-GTCGTCACAATCCACAACCTTATAGT-3' | 5'-TGGGTGTATGGGATTGATGTG AAAGC-3' |
| Tc00g042540 | 5'-TGCCTTCACATCAAAATCATATTTC-3'  5'-CGGAGTTCATCACCATCACTATCA-3' | 5'-TAGCCAGAGCTGATGAATCCC CGGT-3' |
| Tc01g013950 | 5'-TGG AGG TTGATGACCAAAATTTACT-3'  5'-TGCAGAGATTAAGTCATTTGAGCAA-3' | 5'-TGGTGAACGCTCTACCCAGTG TTGGA-3' |
| Tc05g005250 | 5'-TTTGGATGAGTTGGAGAAAGAGA-3'  5'-TAGTCTCCCTCATGGAAGCCTTA-3' | 5’-CAAGGAGTTCTTTGAGGCTTA CACAAGGG-3’ |
| Tc03g009820 | 5'-AGCAATGGCTTCCACCACTT-3'  5'-GGCTGTGAGCTCGATGATGA-3' | 5’-TGCAGCCGTTTCAATGCTTTT GTCTG-3’ |
| Tc09g011440 | 5’-ACTACAGATGCAGGTCTTCTGCCT-3’  5’-TGTTCAGATCTGGCTCAACAGCCT-3’ | 5’-TTTCTTCTTTGTGATTCAACCATG CCTGC-3’ |
| Tc09g033150 | 5’-TTTGTGGAATCGGCCGAAATCTGG-3’  5’-GGAAGTTCTTGCAAGGTCTGTGGT-3’ | 5’-TCGTAATGGACCCGCTGCTATTG CG-3’ |
| Tc04g020270 | 5’-TTTATTAGCCAGTGGGCGACCGTT-3’  5’-AGGTTCATGTCCAAGGATCCACCT-3’ | 5’-CGATCAAGGAACGAACGCGAA GCT-3’ |
